# Timing‐based strategies to minimize the impact of long‐haul travel on sleep: A pilot study in elite athletes traveling for competition

**DOI:** 10.14814/phy2.70654

**Published:** 2025-12-28

**Authors:** Giorgio Varesco, Alix Renaud‐Roy, François Bieuzen, Nathalie Pattyn, Guido Simonelli

**Affiliations:** ^1^ National Institute of Sport of Québec Montréal Quebec Canada; ^2^ Center for Advanced Research in Sleep Medicine, Hôpital du Sacré‐Coeur de Montréal, CIUSSS du Nord de l'Île‐de‐Montréal Montréal Quebec Canada; ^3^ Departement of Medicine Université de Montréal Montréal Quebec Canada; ^4^ Research Unit VIPER Royal Military Academy Brussels Belgium; ^5^ Departement of Neuroscience Université de Montréal Montréal Quebec Canada

**Keywords:** elite performance, jetlag, long‐haul travel, sleep strategy, speed‐skating, zeitgebers

## Abstract

Long‐haul travel poses significant challenges to sleep in elite athletes, yet evidence‐based interventions tested in competitive settings remain scarce. This study investigated the effects of timing‐based interventions on sleep in 10 national‐level Canadian speed skaters prior to a World Cup competition in Beijing (13 time zones crossed). Athletes followed a tailored sleep schedule upon arrival and for the days preceding the competition. Total sleep time in Beijing was not different from Canada (*p* = 0.254) or pre‐season (*p* = 0.999) and was lower the night before travel (*p* < 0.001) due to the early flight to Beijing. When comparing data with a similar dataset presenting no intervention, bedtime was successfully delayed and resulted in later wake‐up time and longer total sleep time. Total sleep time increased by ~10 min/night, suggesting adjustments in sleep–wake rhythm during the first days upon arrival were still present. Race performance was unaffected by travel, with no time effect on overall rank (*p* = 0.74). These preliminary findings suggest that individualized timing‐based strategies might support sleep regulation and circadian re‐synchronization in elite athletes following long‐haul travel. Further studies are warranted to confirm these results in larger samples and explore the effectiveness of customized timing‐based intervention on different time changes and on performance.

## Graphical abstract

Timing‐based strategies for sleep and training scheduling are a promising intervention to increase the total amount of sleep obtained when traveling for competitions.

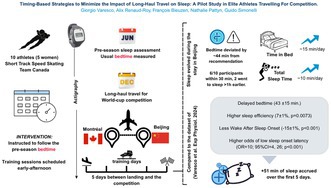

## 1 INTRODUCTION

Long‐haul travels involving around 12 h of time change result in a complete reversal of dark/light cycle, leading to significant circadian rhythm and sleep disturbances (Botonis et al., 2025; Varesco et al., 2024). This particular situation is not unusual in elite athletes, traveling to compete in events such as World Cup races, World Championships, or the Olympic Games. The cost and stress of travel, tight schedule, and competition venue‐specific restrictions, combined with the lack of data on time changes around 12 h, all make it challenging to provide athletes with additional support personnel or implement specific interventions to optimize sleep. Additionally, coaches and athletes might be reluctant to implement new interventions in competition settings, although this is essential for providing recommendations for real‐world scenarios. In a previous study (Varesco et al., 2024), we monitored rest‐activity rhythm using actigraphy data from the Canadian short‐track speed skating national team athletes who traveled across >12 time zones for competitions. Immediately after the long‐haul travel, we noted early bedtimes, likely due to an elevated sleep drive, which gradually re‐adjusted over the next few days. This misalignment resulted in early wake‐up times, and could create conflicts with social, behavioral, and physiological temporal cues, potentially hampering adaptations to the new time zone. Despite all that, sleep was restored after a period of just 5 days. This adaptation can be considered very quick (Eastman & Burgess, 2009), but might not be satisfactory in elite sport: obtaining more sleep upon arrival is crucial for athletes, as they usually need to compete only a few days after the travel.

The most effective set of strategies to improve sleep with jetlag consists of modulating the various zeitgebers contributing to re‐synchronization of circadian rhythms, with a primary role of light, social interactions, and physical activity/training, acting through specific neural and hormonal pathways (Lewis et al., 2018). Emerging scientific consensus and direct evidence highlight the importance of the harmonization of multiple zeitgebers for effective re‐synchronization of circadian rhythms and better sleep (Grabe et al., 2022; Shen et al., 2023). Based on these considerations, timing‐based intervention aiming to regulate the sleep–wake schedule could be a reasonable intervention in competitive contexts. Given the circadian system's inherent tendency to delay sleep, applying westward strategies, such as delaying sleep, might facilitate adaptation when crossing more than eight time zones eastward (Eastman & Martin, 1999; Roach & Sargent, 2019). Delaying bedtime could allow wake‐up time to occur closer to daylight and other social and behavioral cues (e.g., breakfast, morning team meetings etc.). However, providing a bedtime schedule doesn't necessarily imply delayed wake‐up time or improved sleep, and importantly, that athletes would be able to follow it in competitive settings.

Recently, we had the unique opportunity to explore these hypotheses during World Cup competitions in Asia under conditions that closely mirrored that of our previous study (Varesco et al., 2024), with a similar population, destination and training/competition schedule. In this pilot study, athletes were asked to follow an individualized bedtime schedule based on their sleep habits measured during the pre‐season (Varesco et al., 2025). We hypothesize that athletes will be able to follow the sleep schedule, and that delaying bedtime would result in stable sleep in the first few days (up to the first competition) after arrival, as opposed to Varesco et al. (2024).

## 2 METHODS

### 2.1 Participants

Ten short‐track speed skaters from the Canadian National Team (5 females, age: 24 ± 3 years; height: 1.74 ± 0.09 m; body mass: 70 ± 8 kg; Caen Chronotype Questionnaire Eveningness: 42% ± 20%) traveled from Montréal (UTC‐5) to Beijing (UTC+8) for World‐Cup races in December 2024. Athletes did not consume any sleep‐inducing drug, present any declared sleep‐related disturbance/pathology, and did not undergo any specific sleep hygiene education/intervention prior to the study. Athletes provided written consent, and data collection was carried out respecting provincial legislation and conformed to the standard set by the World Medical Association (2013) and was approved by the Ethical Research Committee of the CIUSSS du Nord‐de‐l'Île‐de‐Montréal (n. 2024‐2718).

### 2.2 Study design

Speed skaters' sleep patterns were monitored (actigraphy) for 2 days before and up to 5 days after arriving in Beijing, dividing the travel into three phases: baseline sleep at home, the night before travel, and nights in Beijing. Recommendations were implemented only for the nights in Beijing, while personal initiatives in modifying the sleep schedule in Montreal were discouraged. Athletes' flight for Beijing was scheduled at 7 am (UTC‐5). Athletes traveled in economy class. The travel lasted ~21 h and athletes arrived at the destination at 5 pm (UTC+8; day 0, followed by night 1). The qualifications started on Day 5, with finals from Day 6. We agreed to end the sleep measurements before the finals, as requested by the coaches, because the period of interest was the one separating the landing in Beijing until the start of the competition. We implemented an intervention based on personalized sleep schedules. Bedtime was individualized for each athlete and based on data obtained at the beginning of the pre‐season (i.e., June 2024; Varesco et al., 2025). The pre‐season was identified as the optimal period to measure habitual sleep at home, due to the absence of travel, training camps or competitions. During this period, we monitored the sleep of the speed skaters over a 14‐day period using actigraphy and daily sleep logs (subset of Varesco et al., 2025). The average weekday (Monday–Friday) bedtime for each speed skater was calculated and used for recommendations (Table A1 in Appendix). Athletes were instructed to adhere to the recommended bedtime throughout the period in Beijing. Athletes were also instructed to sleep in as long as possible in the morning with 9 am being the latest to consume breakfast (similarly to Varesco et al., 2024). To promote natural awakening, participants were asked to silence or turn off their phones, avoid setting alarms (except for the 9:00 am breakfast cut‐off), and minimize other potential disturbances. Another factor that was controlled to reflect the conditions of our previous study was the training schedule. This consisted of distributing the main physical activity (i.e., training sessions) in the early afternoon. In our previous study, we proposed that training in the early‐to‐mid afternoon (Varesco et al., 2024) might have facilitated circadian realignment. Indeed, exercising around that time elicits phase shifts comparable to early‐morning exercise and to light exposure (Youngstedt et al., 2019). Thus, in collaboration with the coaches, daily training sessions were scheduled between 1 pm and 3 pm. On Day 2, due to the unavailability of the training facility, training was scheduled at 11 am. This hour was still considered valid based on typical cortisol/melatonin secretion patterns (e.g., Mazzoccoli et al., 2011). Finally, to understand if the long‐haul travel produced systematic impairments in race performance, we retrieved ranking data from the first three World Cup competitions of the season. The first two took place in Montréal, and the third competition was the one analyzed here (Beijing). Data were obtained from the ISU “infostrada” portal (https://isu.html.infostradasports.com).

### 2.3 Actigraphic data

**FIGURE A1:**
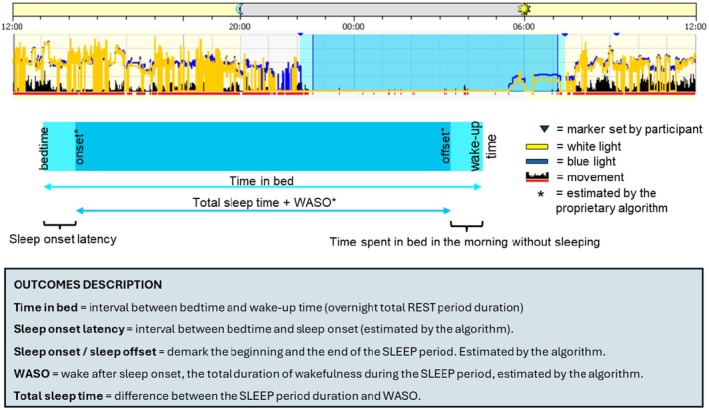
Example of actigraphy data and description of the outcomes. SLEEP and REST interval refers to the wording of the Actiware software interface. Calculations are reported as described in the Actiware user guide: https://www.philips.com/c‐dam/b2bhc/master/sites/actigraphy/solutions/Actiware/actiware‐user‐guide.pdf. In this particular case, a marker was accidentally set at breakfast, visual inspection of the data is important to solve conflicts.

Actigraphic data were collected using a wrist‐worn actigraph (Spectrum Pro, Philips Respironics, Bend, OR) and analyzed using the proprietary software (Actiware 6, Philips Respironics) following similar procedures to our previous work (Varesco et al., 2024), which are detailed in the Appendix. When exporting, local time was manually realigned (i.e., Montréal or Beijing). Actigraphy outcomes were (i) total sleep time, defined as the sleep obtained during the 12 h proximal to midnight (i.e., nighttime sleep), (ii) sleep onset latency, (iii) total duration of the wake after sleep onset (WASO) events, and (iv) sleep efficiency, computed as the percentage of time spent asleep while in bed, compared to the total time spent in bed (de Zambotti et al., 2024; Elbaz et al., 2012). Calculation description for these outcomes is presented in the Appendix (Figure A1). No napping was observed during the stay in Beijing.

### 2.4 Statistical analysis

We assessed adherence to the provided recommendations using linear mixed models and a Bland–Altman plot comparing bedtimes during the pre‐season and the stay in Beijing. Then, we employed linear mixed models or general linear models across three different analyses (for details, see Appendix). First, we compared total sleep time, sleep onset latency, WASO, sleep efficiency, bedtime, and wake‐up time across different phases (i.e., pre‐season, baseline, night before travel, and stay in Beijing). Second, we examined the longitudinal evolution of sleep variables over the first 5 days in Beijing (day effect), using data from our previous work (Varesco et al., 2024) as a control dataset (group effect). Third, we compared competition performance at home (Montreal) versus Beijing, using race ranking as the dependent variable and race distance as a covariate. When a significant main effect or interaction in the linear mixed models was identified, pairwise comparisons were adjusted using Bonferroni correction. WASO was normalized for total sleep time, since for longer nights of sleep it is normal to find longer WASO. The significance threshold was set at *α* = 0.05. All statistical analyses were conducted using R (v4.2.3; R Foundation for Statistical Computing, Vienna, Austria).

## 3 RESULTS

The running code and analyses performed in the present study, with complete model results and verification of assumptions are available at doi: 10.17605/OSF.IO/96QGE.

### 3.1 Characteristics of sleep in pre‐season, at baseline, pre‐travel and in Beijing

Comparison across phases and statistical results from the linear mixed models are presented in Table 1.

**TABLE 1:** Sleep data for each phase of the study.

	Pre‐season	Baseline	Pre‐travel	Beijing	Main effect
Total sleep time (min)	485 ± 46	453 ± 67	262 ± 62^+++,###^	493 ± 76^$$$^	*p* < 0.0001
Sleep efficiency (%)	89 ± 5	88 ± 5	88 ± 8	89 ± 8	*p* = 0.819
Sleep onset latency (min)*	23 ± 19	18 ± 23	20 ± 24	12 ± 11	*p* = 0.082
WASO (min)	29 ± 9	25 ± 7	14 ± 9^+++,#^	24 ± 10^+,$^	*p* < 0.0001
Relative WASO (% total sleep time)	5.9 ± 1.6	5.5 ± 1.3	5.4 ± 3.1	5.1 ± 2.8	*p* = 0.258
Time in bed (min)	546 ± 46	513 ± 72	297 ± 63^+++,###^	551 ± 56^$$$^	*p* < 0.0001
Bedtime (hh:mm)	22:40 ± 0:41	23:07 ± 01:07	22:35 ± 01:01	22:06 ± 00:38^+++,###^	*p* < 0.0001
Wake‐up time (hh:mm)	7:46 ± 00:42	07:40 ± 00:56	03:32 ± 00:11^+++,###^	07:16 ± 00:44^+++,$$$^	*p* < 0.0001

*Note*: Data are presented as mean ± SD. ^+^different from preseason: ^+^ *p* < 0.05, ^++^ *p* < 0.01, ^+++^ *p* < 0.001; ^#^different from baseline: ^#^ *p* < 0.05, ^##^ *p* < 0.01, ^###^ *p* < 0.001; ^$^different from pre‐travel night: ^$$$^ *p* < 0.001 and *when sleep onset latency > 1 min (see Appendix).

Abbreviation: WASO, wake after sleep onset.

Total sleep time and time in bed were reduced the night before travel (Pre‐travel) compared to all other phases (phase effects: all *p* < 0.0001). WASO was the highest in pre‐season the lowest in pre‐travel (phase effect: *p* < 0.0001, Table 1), but these differences disappeared normalizing WASO to total sleep time (phase effect: *p* = 0.258). Wake‐up time was set earlier pre‐travel due to the early flight (*p* < 0.001). In Beijing, athletes were going to bed and woke up earlier compared to pre‐season (*p* < 0.001; Table 1, see Adherence to the recommendations). Sleep efficiency and sleep onset latency showed no significant changes across phases (*p* = 0.819 and *p* = 0.082, respectively). However, sleep onset latency exhibited zero‐inflation and a right‐skewed distribution. Thus, a two‐part hurdle model was implemented (Appendix). When evaluating the probability of sleep onset latency being zero (immediate sleep), the likelihood ratio test indicated a significant main effect for phase (*p* < 0.001). Compared to pre‐season, the odds of immediate sleep were higher at baseline (OR = 8; 95% CI = 2, 37, *p* = 0.0076), pre‐travel night (OR = 8; 95% CI = 2, 37, *p* = 0.0076), and in Beijing (OR = 7; 95% CI = 3, 30, *p* < 0.001). When sleep onset latency was >1 min, no main effect of phase was observed (Table 1).

### 3.2 Evolution of sleep in Beijing

Time in bed showed a day effect (*p* < 0.0001), without a main effect of group (*p* = 0.1354) or group × day interaction (*p* = 0.3906), suggesting that time spent in bed (I ± SE = 498 ± 10 min) was similar and increased similarly across days in both groups. Specifically, time in bed increased by ~15 min/day (*β* ± SE = 14 ± 2min/day).

**FIGURE 1:**
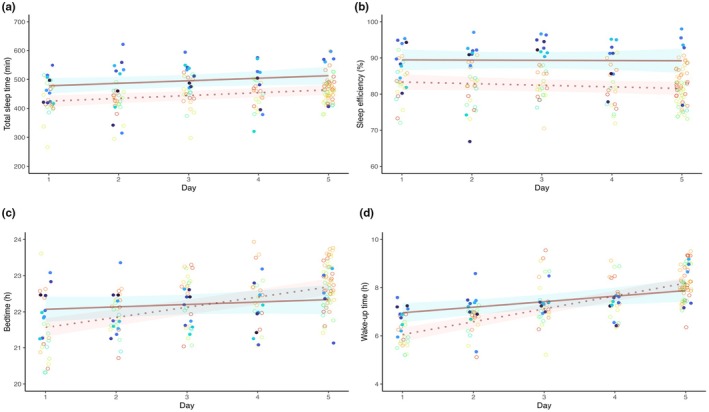
Evolution of total sleep time (a), sleep efficiency (b), bedtime (c), and wake‐up time (d) in the 5‐day following the arrival in Beijing that preceded the first competition. Solid circles (blue shading; *n* = 10) represent the speed skaters from the intervention group. Empty circles (*n* = 19) represent participants from Varesco et al. (2024). Note that data from Varesco et al. (2024) contain more points at “Day 5” as additional nights occurred before the competition in 2017, and presented so to keep model estimate and presentation consistent across studies. Colors are individual for each speed skater. For each plot, the brown lines and shades represent the linear mixed model estimates ± SE of the present study (solid line) and Varesco et al., 2024 dataset (dotted line).

For total sleep time, we found a main effect of day (*p* = 0.0004) and group (*p* = 0.0067) without a group × day interaction (*p* = 0.8646; Figure 1a). Total sleep time (I ± SE = 416 ± 11 min) increased across days by ~10 min/day (*β* ± SE = 9.56 ± 2.37 min/day). Speed skaters in the present study slept overall for a longer time compared to the control dataset (*β* ± SE = 51.19 ± 10.33 min).

Sleep efficiency showed a group effect (*p* = 0.0073), without a day effect (*p* = 0.1192) or group × day interaction (*p* = 0.5384, Figure 1b). Speed skaters showed overall higher sleep efficiency compared to the control dataset (I ± SE = 82 ± 1%; *β* ± SE = 7 ± 1%).

WASO (normalized to total sleep time) showed a group effect (*p* < 0.001), without a day effect (*p* = 0.647) or a group × day interaction (*p* = 0.224, Figure 1b). Speed skaters showed overall lower WASO normalized to total sleep time compared to the control dataset (I ± SE = 20 ± 1%; *β* ± SE = ‐15 ± 1%).

A two‐part hurdle model was implemented for sleep onset latency (see Appendix). We found a group effect (*p* < 0.0001), with speed skaters showing higher odds of immediate sleep onset compared to the control dataset (OR = 10; 95% CI = 4, 26), There was no day effect (*p* = 0.878) and no day × group interaction (*p* = 0.862). When sleep onset latency was >1 min, we observed a day effect (*β* ± SE = 0.115 ± 0.044; *p* = 0.0096). There was also a group effect, with longer onset latencies in the present study group compared to the control dataset (*β* ± SE = 0.537 ± 0.229; *p* = 0.0189).

### 3.3 Adherence to the recommendations

For bedtime, we observed a group × day interaction (*p* = 0.0049, Figure 1c). Bedtime (I ± SE = 21.29 ± 0.16 h) was progressively delayed across days in the athletes of the control dataset (*β* ± SE = 0.28 ± 0.03 h/day), whereas this delay was reduced in athletes of the present study (*β* ± SE = −0.21 ± 0.08 h/day). Speed skaters in the intervention group also showed overall later bedtimes compared to the control dataset (*β* ± SE = 0.72 ± 0.25 h). Similarly, wake‐up time showed a group × day interaction (*p* = 0.0009, Figure 1d). In the control dataset, wake‐up time (I ± SE = 5.52 ± 0.18 h) was progressively delayed across days (*β* ± SE = 0.53 ± 0.04 h/day), and this delay was attenuated in athletes of the present study (*β* ± SE = –0.31 ± 0.09 h/day). Overall, speed skaters woke up later compared to the control dataset (*β* ± SE = 1.21 ± 0.30 h).

**FIGURE 2:**
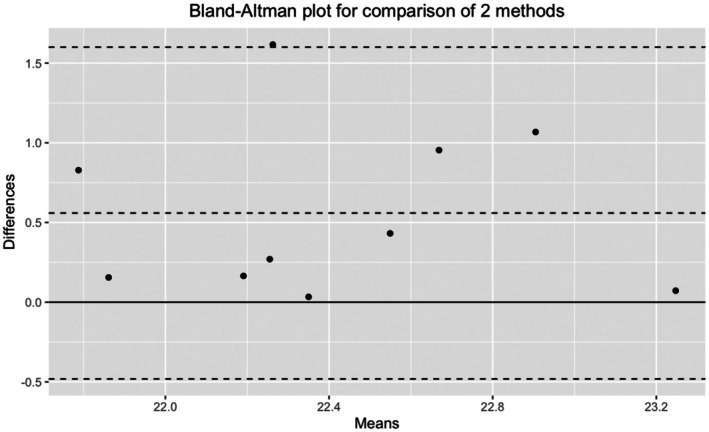
Bland–Altman plot comparing the recommended bedtime provided and average bedtime observed in Beijing. The *X* axis shows the mean of the two bedtimes (recommendation and implementation), and the *Y* axis represents their difference for each speed skater. The dotted line indicates the mean difference (bias) with 95% limits of agreement. A solid line was plotted at zero difference for reference.

When comparing the average bedtime in Beijing with the recommendation (Appendix) the model estimated a 44 ± 14 min difference (*β* ± SE, *p* = 0.0016). This difference did not change across days during the stay in Beijing (*p* = 0.227). The Bland–Altman plot (Figure 2) indicates that six among 10 participants kept bedtime within 30 min of the schedule, while two athletes went to sleep >1 h before the recommended time.

### 3.4 Race performance

**FIGURE 3:**
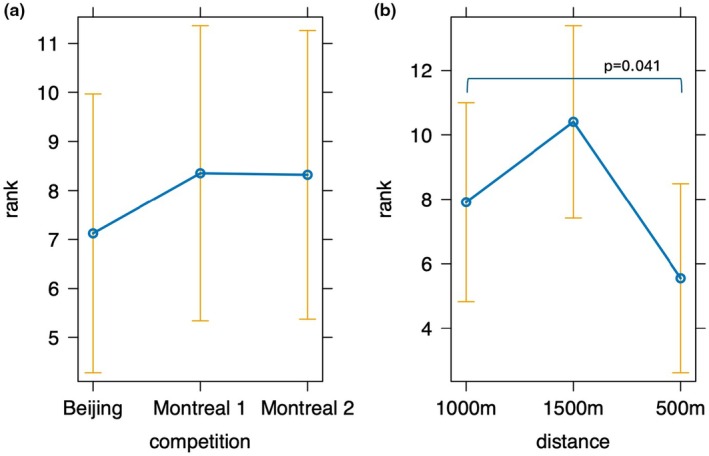
Estimated marginal effects of competition and distance on performance rank ± 95% CI. A difference was found between 500 and 1500 mt competition distance.

No effect of competition was found on the overall rank (*p* = 0.74; Figure 3a), while an effect of distance on rank was found (*p* = 0.044). Ranking at the 500 m was overall better compared to 1500 m, while no difference was found between 500 m and 1000 m or 1000 m and 1500 m (Figure 3b).

## 4 DISCUSSION

This study is the first to attempt the implementation of a timing‐based intervention in competitive contexts to improve sleep in elite athletes after long‐haul travels. Our intervention aimed to stabilize sleep timing from the first night following long‐haul travel, and to delay wake‐up time to enhance the influence of local time cues in promoting circadian resynchronization. Following recommendations, we observed two main results: first athletes overall were unable to follow them, contradicting our hypotheses. Bedtime and wake‐up time were more stable across days compared to our previous study, as shown by a group × day interaction, but time in bed or total sleep time increased across days during the stay in Beijing, as observed in our previous study (Varesco et al., 2024). The second result, which might be related to the intervention, is that despite similar time in bed compared to (Varesco et al., 2024), more sleep was obtained in the present study during the stay in Asia, driven by shorter sleep onset latency and WASO, improving sleep efficiency.

None of the sleep‐related outcomes differed between the pre‐season and couple of days before travel to Beijing (Baseline). This result might be driven by the lower number of nights available for Baseline (*n* = 2). However, it also confirms that athletes did not (consciously or unconsciously) implement strategies to prepare for the travel (e.g., sleep banking). Less sleep was obtained between the night before travel and the other phases, due to the early flight schedule. This, combined with the inability to detect reliably a clear sleep phase during the ~21 h travel could indicate the presence of sleep debt (and possible travel‐related fatigue) upon arrival, resulting in a high perceived drive to sleep.

Upon arrival in Beijing, athletes were unable to follow the recommended bedtime and this could be due to the combination of difficulties in resisting the perceived drive to sleep and a reduction in the number of Zeitgebers (e.g. light, social activities) in the evening, slowing phase‐shift and circadian realignment. Despite early‐afternoon exercise, which could have a phase‐advancing effect (Youngstedt et al., 2019), we cannot exclude that exercise timing also had an impact on increasing perceived sleep drive in the evening. Finally, shared bedrooms could be a third factor explaining the inability to follow recommendations, that is, athletes sharing a room would tend to have a similar lights‐off time.

The direct comparison with the dataset of Varesco et al. (2024) helps clarify the effects of the intervention and its limitations. Using the models' predicted values and assuming comparable datasets, our intervention led to a more consistent sleep schedule across days in Asia. Indeed, in the present study the changes in bedtime and wake‐up hours (delayed by ~4 and ~11 min/night, respectively) were lower than the previous dataset (~17 and ~30 min/night; Varesco et al., 2024). Importantly, we observed a similar time in bed for both datasets, but an overall 51 min of additional sleep for the first 5 days in Asia in the present study. This was driven by the higher sleep efficiency, higher odds to fall asleep immediately after bedtime, and lower WASO compared to the dataset of Varesco et al. (2024), possibly due to the higher sleep drive caused by postponing bedtime. Despite being promising, these results need additional experimental confirmation.

One main result is that total sleep time evolved similarly in both studies during the stay in Asia, with a similar percentage of WASO duration and sleep onset latency across days or changing very slightly. Adjustments in wake–sleep schedules following long‐haul travel could rarely occur in a few nights, especially with a complete reversal of the day/night cycle. Despite the need for modifications to the intervention, as athletes were unable to follow recommended bedtimes in the present study, these preliminary results show that delaying bedtime might represent a simple strategy to improve sleep and could encourage future studies evaluating the effect of timing‐related strategies in competitive contexts. Time‐based strategies are promising and could maximize circadian synchronization if combined with strategies that are more popular in the literature, such as light exposure or melatonin supplementation. The specific phase‐advancing effect of training could also be further considered in future investigations (Varesco et al., 2024; Youngstedt et al., 2019).

Improved sleep patterns do not necessarily translate to enhanced physiological performance. The 2024 World Cup calendar provided a natural experimental setting, with two home competitions (Montréal) followed shortly by one in Beijing. Performance outcomes were not consistently lower in Beijing, aligning with our previous findings (Varesco et al., 2024), and suggesting limited travel‐related impact on race performance. A few aspects should be considered when interpreting these performance outcomes. First, we do not have information on other teams. We cannot exclude the possibility that opponents have experienced varying levels of jet lag or adopted countermeasures (e.g., light exposure, pharmacological aids). Second, factors such as nutrition, environmental conditions, or access to recovery modalities might differ between Montreal and Beijing, impacting the performance or well‐being of teams and individuals differently. Third, the absence of a fully controlled baseline limits causal inference.

### 4.1 Limitations

Because the invasiveness of the measure is a priority when testing athletes traveling for World Cup competitions, several methodological limitations apply to this study. For instance, no direct measures of circadian phase or subjective jet lag symptoms were collected, preventing us from determining the extent of circadian misalignment. We did not collect qualitative and quantitative data such as perceived sleep quality, caffeine consumption, or bedding comfort that would allow more in‐depth analyses. The sample size of this study is small. However, it included a rare population of top‐level speed skaters competing at the World Cup level, representing the highest standards in sport. Implementing a control group was not feasible due to the limited number of athletes, potential behavioral cross‐contamination, logistical constraints (e.g., shared training times and bedrooms), and ethical concerns given the potentially performance‐enhancing nature of the intervention. Nevertheless, we are convinced that data from our previous study represented a valid control dataset, as the study was conducted in a similar population and context, and analyzed using the same procedures by the same research team (Varesco et al., 2024).

## 5 CONCLUSIONS

Overall, these preliminary findings suggest that timing‐based strategies for sleep and training schedule are a promising intervention to increase the total amount of sleep obtained when traveling for competitions, possibly helping elite athletes to resynchronize their circadian rhythm more quickly. However, difficulty in following recommendations and lack of mechanistic data highlight the need for further research on this topic. These findings are an important step in understanding the evolution of sleep following long haul travels involving a complete reversal of the day/night cycle, and towards a validated set of tools available to people to sleep well after long‐haul travels.

## AUTHOR CONTRIBUTIONS

All authors contributed to the design of the study. GV and ARR conducted the experiments. GV performed data analysis, performed statistical analysis and wrote the first draft of the manuscript. FB, NP and GS contributed to results interpretation and corrected the manuscript. All authors have read and approved the final version of this manuscript and agree to be accountable for all aspects of the work in ensuring that questions related to the accuracy or integrity of any part of the work are appropriately investigated and resolved. All persons designated as authors qualify for authorship, and all those who qualify for authorship are listed.

## FUNDING INFORMATION

This work was supported by the PRIDI program from the National Institute of Sport of Quebec (PRIDI‐76), the Ministère de l'Enseignement Supérieur du Quebec through the University of Montreal Parteneriat‐UdeM program, and from the Research Center of the CIUSSS NIM. GV was supported by a Mitacs Accelerate Fellowship (IT42957). GS was supported by the FRQS Research Scholar Program (#297725).

## CONFLICT OF INTEREST STATEMENT

None.

## ETHICS STATEMENT

Data collection was carried out respecting provincial legislation and conformed to the standard set by the World Medical Association (2013) and was approved by the Ethical Research Committee of the CIUSSS du Nord‐de‐l'Île‐de‐Montréal (n. 2024‐2718). All participants provided written consent before the study.

## Data Availability

The research data will be kept confidential until the Olympic Games 2026 to not compromise ethical standards and legal agreements between the Institut National du Sport du Québec and Speed Skating Canada. All codes and results can be found at doi: 10.17605/OSF.IO/96QGE.

## ADDITIONAL METHODOLOGICAL INFORMATION

### ACTIGRAPHY

Athletes wore a Spectrum Pro (Philips Respironics, Bend, OR) actigraph on their non‐dominant wrist. The device recorded data over 30‐s epochs, as we were interested in sleep–wake behavior, rather than energy expenditure. The threshold of sensitivity for sleep was set high using the proprietary software (Actiware 6, Philips Respironics). Previous research showed that these settings yielded good agreement, specificity, and sensitivity in estimating total sleep time compared to polysomnography in elite athletes (Sargent et al., 2016). Elite speed skaters were familiar with wearing actigraphy monitors and were instructed to avoid covering the light sensor with clothes around bedtime and wake‐up time. Bedtime and wake‐up time were defined as the “subjectively reported behavioural indicators reflecting the time (hh:mm:ss) a person chooses to start trying to fall asleep and conclude their attempts to sleep, respectively” (de Zambotti et al., 2024). Skaters were instructed to press the event marker button on the device at both bedtime and wake‐up time. This button sets a temporal marker when exporting data. The temporal marker and light sensor data were used to verify the software's algorithm estimation of bedtime and wake‐up time. The description of all outcomes exported from actigraphic analysis is detailed in Figure A1. Sleep efficiency was calculated as the percentage of time spent asleep while in bed compared to the total time spent in bed (de Zambotti et al., 2024; Elbaz et al., 2012). Since we did not observe daytime naps in the present data, total sleep represents sleep obtained during nighttime. Data collected during travel were excluded because absolute timestamps were unreliable for determining bedtime and wake‐up time (i.e., data during the ~21 h of travel were unusable). Five participants (S2, S3, S5, S6, and S7) wore the watch also on night 5. All data available were used for analyses. Recommendations indicate the average bedtime during the weekdays in preseason and are presented in the Table A1, together with the missing data of the present study.

**TABLE A1:** Recommended bedtime (hh:mm) provided to each participant and number of nights recorded.

Id	Recommendation	Nights recorded in Beijing	Notes
S1	21:55	4	
S2	22:50	5	
S3	22:20	4	Forgot to wear at night 4
S4	23:20	3	Forgot to wear at night 3
S5	22:25	5	
S6	22:10	5	
S7	22:25	5	
S8	23:25	4	
S9	22:05	4	
S10	23:05	4	

### STATISTICAL ANALYSIS

For all analyses, linear mixed‐effects models [lme4 package] were fitted to the data using the restricted mean likelihood method. For all models, analysis of deviance (Type III Wald χ^2^ tests) was used to evaluate the global main effects of the phase or day for the first and second analyses, respectively [car package]. The *p* values were extracted from *F*‐tests using Satterthwaite's degrees of freedom method [lmerTest package]. A random intercept for participants was inserted into each model. The empirical test of the model assumptions was performed via model residuals graphical analysis of the Q‐Q plots. Emmeans package was used for post‐hoc analysis. Estimates from the models and standard errors were calculated using the least‐squares means method.

Sleep onset latency exhibited zero‐inflation and a right‐skewed distribution. To avoid log‐transformation that could impact interpretability, a two‐part hurdle model was implemented. First, we used a logistic mixed‐effects model to estimate differences across phases in the probability of sleep onset latency being zero (glm1). Second, a generalized linear mixed‐effects model assuming a Gamma distribution with a log link was used to model sleep onset latency duration (glm2). Both models were fit using the lme4 and glmmTMB packages in R. Simulated residuals [DHARMa package] were used when testing residual distribution for the glmmTMB, and a Wald test (likelihood ratio test) was used to assess the main effect of phase, comparing the fitted model with a null model.
